# Ultra‐processed food addiction in a nationally representative sample of older adults in the USA

**DOI:** 10.1111/add.70186

**Published:** 2025-09-29

**Authors:** Lucy K. Loch, Matthias Kirch, Dianne C. Singer, Erica Solway, J. Scott Roberts, Jeffrey T. Kullgren, Ashley N. Gearhardt

**Affiliations:** ^1^ Department of Psychology University of Michigan Ann Arbor MI USA; ^2^ Institute for Healthcare Policy and Innovation University of Michigan Ann Arbor MI USA; ^3^ Department of Health Behavior and Health Equity, School of Public Health University of Michigan Ann Arbor MI USA; ^4^ Center for Clinical Management Research, VA Ann Arbor Healthcare System Ann Arbor MI USA; ^5^ Departments of Internal Medicine and Health Management and Policy, Schools of Medicine and Public Health University of Michigan Ann Arbor MI USA

**Keywords:** mental health, older adults, physical health, social isolation, ultra‐processed foods, ultra‐processed food addiction

## Abstract

**Aims:**

Ultra‐processed foods (UPFs; industrially produced foods typically containing unnaturally elevated levels of refined carbohydrates and/or added fats) became more widely introduced into the United States (US) food environment in the 1980s and have proliferated since. UPFs have been shown to trigger an addictive‐like response. This study examines the prevalence of ultra‐processed food addiction (UPFA) in older US adults and its association with various health domains.

**Design:**

In July 2022, a cross‐sectional online and telephone survey was conducted using the University of Michigan National Poll on Healthy Aging (NPHA). Gender‐stratified analyses examined the association between UPFA and perceptions of physical and mental health, and social isolation. Prevalence ratios were calculated, unadjusted and adjusted for age, race/ethnicity, education, and income.

**Setting:**

Nationally representative sample of older adults (aged 50–80 years) in the United States.

**Participants:**

The sample included 2038 older adults (49.4% aged 50–64 years and 50.6% aged 65–80 years, 51.2% women, M age = 63.6, standard deviation = 8.1).

**Measurements:**

The modified Yale Food Addiction Scale 2.0 (validated measure that applies the diagnostic criteria for substance use disorder to the overconsumption of UPFs) was used to assess diagnostic criteria for UPFA. Various self‐reported items were used to assess health‐related domains (i.e., physical and mental health, social isolation).

**Findings:**

The overall prevalence of UPFA was 12.4%, higher among women (16.9%) than men (7.5%), with the highest rate in women aged 50–64 (21%). Men reporting being overweight were 19.14 (95% confidence interval [CI] [5.26–69.66]) times more likely to meet the criteria for UPFA. Women reporting being overweight were 11.44 (95% CI [4.56–28.71]) times more likely to meet UFPA criteria. Women and men reporting worse physical health were 1.93 (95% CI [1.26–2.98]) times and 2.99 (95% CI [1.70–5.26]) times more likely to meet the criteria for UPFA, respectively. Similarly, women reporting worse mental health were 2.78 (95% CI [1.79–4.32]) times more likely to meet the criteria for UPFA, with men 4.02 (95% CI [2.19–7.38]) times more likely. Lastly, women and men reporting feelings of social isolation were 3.40 (95% CI [2.16–5.34]) times and 3.35 (95% CI [1.83–6.14]) times more likely to meet UFPA criteria.

**Conclusion:**

Ultra‐processed food addiction appears to be prevalent among older adults in the United States, particularly among women who were in adolescence and early adulthood when the nutrient quality of the US food supply worsened. Addictive patterns of UPF intake appear to be associated with poorer physical health, mental health, and social well‐being.

## INTRODUCTION

The modern food environment in the US is currently dominated by ultra‐processed foods (UPFs). Many industrially mass‐produced processed foods have unnaturally elevated levels of refined carbohydrates and/or added fats, and are classed as UPFs [1]. Today it is estimated that 60% of the total energy intake comes from UPFs in adults [2]. The 1970s and 1980s have been identified as a key period when UPFs became more common in the US food supply [3, 4], and the degree of added sugars and saturated fats also rose rapidly during this time [5, 6, 7]. This increase in UPFs coincides with the increased involvement of tobacco companies in developing, producing and marketing UPFs [8]. The food manufacturers that were owned by tobacco companies created foods with hyperpalatable ingredients, which has now spread across the modern food supply [9]. Individuals who are now older adults were in developmentally sensitive stages (i.e. childhood, adolescence and early adulthood) during the 1970s and 1980s, precisely when tobacco‐owned food manufacturers were shaping the market with addictive UPFs [9]. Early exposure to these products may have shaped enduring consumption patterns among today’s older adults. While the tobacco industry sold off their food manufacturer holdings in the mid‐2000s, their practices instituted in the 1970s and 1980s are still used today and have left an indelible impact on our food supply.

Most UPFs are designed to be highly rewarding through combinations of palatable ingredients and additives that enhance flavor and texture [10]. The hyper‐rewarding nature of UPFs may make them capable of triggering addictive mechanisms that can increase the propensity for compulsive patterns of intake (i.e. loss of control over intake, intense craving, continued use despite negative consequences) [3, 10]. The Yale Food Addiction Scale (YFAS) is the most common measure of UPF addiction (UPFA) [11, 12]. The YFAS applies the diagnostic criteria for substance use disorders (SUDs) (e.g. loss of control over intake, intense craving, continued use despite negative consequences, withdrawal, tolerance) to the intake of common UPFs (e.g. chocolate, salty snacks, sugar‐sweetened beverages) [13]. While UPFA is not currently recognized as an official diagnosis, research on this condition has been growing substantially in the past 20 years. A meta‐analysis of over 200 studies from around the world estimated the prevalence rate for UPFA to be at 14% for adults based on the YFAS, which is similar to the prevalence rates of other addictive substances such as alcohol and tobacco (14% and 18%, respectively) [12]. As anticipated, UPFA is associated with lower diet quality, a greater intake of UPFs, and a lower intake of fruits and vegetables [14, 15, 16]. The presence of UPFA has also been linked with physical health concerns, such as overweight and obesity [11, 12, 17, 18] and diet‐related diseases like diabetes [19, 20]. Worse mental health has also consistently been associated with UPFA, including increased high‐risk substance use, depression, anxiety and traumatic stress disorders [12, 21, 22, 23, 24].

Older adults (aged 50–80 years) were in a critical developmental period during the 1970s and 1980s when UPFs became more widely introduced into the American food environment. During the 1970s and 1980s older adults in the current study were 8–38 years old when the food environment shifted. Early adolescence through to early adulthood are high‐risk developmental periods for addiction, owing to heightened reward sensitivity, impulsivity and emotional dysregulation [25, 26]. Older adulthood is also an important development period where the lifetime effects of cumulative health behaviors, such as overconsumption of UPFs, can manifest [27, 28]. Yet, most research on UPFA has focused on early and middle adulthood [12].

To our knowledge, only one prior study has focused on UPFA in older adults. In this one prior study, which used a large epidemiological sample of female nurses, UPFA was associated with an 18‐fold increase in the likelihood of having a body mass index (BMI) of ≥35 kg/m^2^ in women aged 62–88 years [29]. Furthermore, UPFA was also associated with an increased risk of hypercholesterolemia and depression in these same women [29]. However, this sample was limited by only including female nurses, being more educated and less racially and socio‐economically diverse than nationally representative samples [29]. This study also did not investigate social isolation, which has been identified as an increasingly important issue for older adults and is implicated in worse mental health and problematic substance use [30, 31, 32, 33].

There has also been no scientific investigation of potential gender differences in UPFA in older adults. Gender differences in other addictive disorders such as alcohol use disorder (AUD) and tobacco use disorder (TUD) in older adults are marked, with older adult men having higher prevalence rates [34]. However, older women appear to have higher rates of binge eating and eating disorder symptoms [35, 36]. Prior research in predominantly early and middle adulthood has been mixed regarding gender differences in UPFA [11, 12]. Thus, it is crucial to investigate how UPFA may present in older adult men as well as women to identify potential gender differences.

In the current study, we investigated the prevalence of UPFA in adults aged 50 years and older in a sample recruited to be nationally representative of the US (University of Michigan National Poll on Healthy Aging; *n* = 2038). Prevalence was explored both by age cohort (ages 50–64 years relative to ages 65 years and older) and gender. Next, we explored the association of UPFA with self‐reported physical, mental and social well‐being in these older adults. Finally, analyses were gender‐stratified to identify potential gender differences in UPFA and its association with these health‐related measures.

## METHODS

### Study design

A cross‐sectional study was conducted by the University of Michigan National Poll on Healthy Aging (NPHA). The NPHA is a recurring survey of US adults aged 50 years and older on health, healthcare and health policy issues. This poll is designed to capture a broad snapshot of health and functioning using brief assessments to minimize participant burden. The University of Michigan Institutional Review Board reviewed the NPHA and deemed it exempt and not requiring informed consent.

### Participants

In July 2022, during the COVID‐19 pandemic, we surveyed a nationally representative sample of older adults aged 50–80 years (*n* = 2163; completion rate 68.6%) through the NPHA using the NORC (previously the National Opinion Research Center) AmeriSpeak® panel. This panel is a probability‐based sample designed to be representative of the US household population. Respondents who did not complete all items of the modified YFAS (mYFAS 2.0) were excluded from the analyses (*n* = 114; final *n* = 2038). Demographic data including age, gender, race and ethnicity, household income, and education level were self‐reported in the AmeriSpeak® panel data and are presented in Table 1. Overall, the average age in the sample was 63.6 years, with 51.2% women, 69.8% White, 45.7% with a household income below $60,000 and 35.2% with a bachelor’s degree or higher.

**TABLE 1:** Sample demographics.

	Respondent gender			
	Men (*n* = 994)		Women (*n* = 1044)	
	Weighted %	95% CI	Weighted %	95% CI
**Demographic cariables**				
Age – two categories				
50–64 years	58.1	(54.0–62.1)	54.7	(50.6–58.8)
65–80 years	41.9	(37.9–46.0)	45.3	(41.2–49.4)
Race/ethnicity				
White, non‐Hispanic	71.2	(66.9–75.1)	68.4	(64.2–72.4)
Black, non‐Hispanic	9.1	(6.8–12.1)	12.5	(9.9–15.7)
Hispanic	12.4	(9.9–15.4)	12.7	(10.2–15.6)
Other, non‐Hispanic	7.3	(4.8–11.0)	6.4	(4.0–10.1)
Education				
High school or less	36.8	(32.5–41.4)	42.0	(37.7–46.4)
Some college	25.8	(22.9–28.8)	24.9	(22.0–27.9)
Bachelor’s degree or higher	37.4	(33.5–41.4)	33.1	(29.4–37.1)
Four‐level household income				
Less than $30,000	18.9	(15.6–22.7)	22.0	(18.7–25.7)
$30,000 to <$60,000	23.2	(20.0–26.6)	27.0	(23.7–30.7)
$60,000 to <$100,000	24.3	(20.9–28.2)	29.2	(25.5–33.3)
$100,000 or more	33.6	(29.8–37.6)	21.7	(18.3–25.5)
**Predictor variables**				
How would you describe your weight?				
Underweight	1.2	(0.5–2.8)	0.9	(0.4–1.7)
Slightly underweight	7.1	(5.1–9.9)	3.6	(2.5–5.1)
About the right weight	27.0	(23.5–30.9)	24.0	(20.6–27.8)
Slightly overweight	38.8	(34.8–43.0)	40.1	(36.1–44.3)
Overweight	25.8	(22.5–29.5)	31.4	(27.5–35.6)
Physical health				
Excellent/very good/good	75.2	(71.4–78.6)	80.8	(77.1–83.9)
Fair or poor	24.8	(21.4–28.6)	19.2	(16.1–22.9)
Mental health				
Excellent/very good/good	87.7	(84.4–90.4)	90.0	(87.1–92.4)
Fair or poor	12.3	(9.6–15.6)	10.0	(7.6–12.9)
In the past year, how often have you felt isolated from others?				
Hardly ever	66.7	(62.7–70.5)	56.5	(52.2–60.7)
Some of the time/often	33.3	(29.5–37.3)	43.5	(39.3–47.8)

### Measures

#### mYFAS 2.0

The mYFAS 2.0 is a validated 13‐item measure of addictive responses to UPFs that reflects the current diagnostic criteria for SUDs. This measure assesses the frequency of behaviors like loss of control over intake, intense cravings, withdrawal, tolerance and continued use despite negative consequences when consuming common UPFs, such as chocolates, ice cream, French fries and pizza, within the past year. The mYFAS 2.0 provides a scoring threshold for each of the 11 diagnostic criteria for an SUD in the context of UPF intake and a threshold for UPFA based on the criteria for an SUD diagnosis (i.e. two or more symptoms plus clinically significant impairment/distress). The mYFAS 2.0 has been found to have high reliability, discriminant validity and convergent validity [37], and in this present study had high internal consistency (with a Kuder–Richardson coefficient of KR = 0.82).

#### Weight status

One item assessed the weight status of participants: ‘How would you describe your weight status?’ Responses were given on a five‐point Likert scale that included underweight, slightly underweight, about the right weight, slightly overweight and overweight.

#### Physical health

Physical health was assessed by one item where participants were asked, ‘In general, how would you rate your physical health?’ A five‐point Likert scale response was used, with responses ranging from poor to excellent. Prior research has found this item to be predictive of greater morbidity and earlier mortality [38, 39, 40].

#### Mental health

One item assessed mental health: ‘In general, how would you rate your mental health?’ Responses were presented on a five‐point Likert scale ranging from poor to excellent. This single‐item measure of mental health has been associated with various multi‐item measures of mental health, self‐reported health status and health conditions [41].

#### Social isolation

Social isolation was measured with one item from the University of California, Los Angeles (UCLA) Loneliness Scale, a validated measure of loneliness [42]. Participants were asked, ‘In the past year, how often have you felt isolated from others?’ Responses were presented on a three‐point Likert scale (hardly ever, some of the time or often). This one‐item measure of social isolation has been associated with fair or poor mental and physical health, and those with health problems or activity‐limiting disability [43].

### Data analysis

The analyses conducted in the current study are exploratory and thus not pre‐registered. First, the percentage of older adults in the current sample who met the diagnostic criteria for UPFA was calculated. Second, gender differences in the weighted proportions of individual UPF diagnostic criteria and UPFA were investigated using Pearson chi‐square tests. Next, associations of UPFA with demographic and predictor variables were evaluated using Pearson chi‐square tests in gender‐stratified analyses. *Post hoc* comparisons were completed using logistic regression with pairwise comparisons of marginal linear predictors using the Bonferroni adjustment. Finally, Poisson regression analysis was used to estimate adjusted prevalence ratios (PRs) for gender‐stratified relationships among perceived physical health, mental health, social well‐being and UPFA. Additionally, race/ethnicity, income and education were chosen as covariates given their association with the predictor variables and UPFA in prior research [44, 45, 46, 47, 48, 49]. Poisson regression is a well‐regarded method of estimating rate ratios [50, 51]. The Poisson method was compared with a logistic regression and a log‐binomial model. As results were consistent across all models and reporting rate ratios was of importance, the results of the Poisson regression are reported below. These same analyses were repeated without gender stratification to get total sample results, which are presented in the supporting information (Tables S1–S4). Two‐sided *P* < 0.05 was considered statistically significant. The regression results are reported both unadjusted and adjusted for age, race and ethnicity, education and income. All analyses were completed using Stata 18 (StataCorp LLC, College Station, TX, USA).

## RESULTS

### UPFA symptom endorsement overall and by gender

The overall prevalence rate of UPFA among older adults in this sample was 12.4% (Table S2). Women (16.9%) endorsed higher levels of UPFA relative to men (7.5%). Women also had significantly higher levels of certain symptoms of UPFA (e.g. greater cravings, inability to cut down on intake) and impairment/distress (Table 2).

**TABLE 2:** Response frequencies meeting ultra‐processed food addiction (UPFA) diagnostic criteria measured by mYFAS 2.0.

Symptom	Threshold	Respondent gender				*P*
		Men meeting threshold		Women meeting threshold		
		Proportion	95% CI	Proportion	95% CI	
I had such strong urges to eat certain foods that I could not think of anything else	Once a week	18.2	(14.9–22.0)	29.1	(25.3–33.2)	<0.001
I tried and failed to cut down on or stop eating certain foods	2–3 times a week	15.6	(12.6–19.1)	23.5	(19.9–27.5)	0.002
If I had emotional problems because I had not eaten certain foods, I would eat them	Once a week	12.6	(10.0–15.8)	21.3	(18.1–25.0)	<0.001
Eating the same amount of food did not give me as much enjoyment as it used to	2–3 times a week	9.9	(7.8–12.7)	15.9	(12.8–19.6)	0.004
I kept eating in the same way even though my eating caused emotional problems	Once a month	13.3	(10.3–16.9)	9.6	(7.4–12.4)	0.074
My friends or family were worried about how much I overate	Once a month	8.9	(6.8–11.7)	11.2	(8.6–14.5)	0.231
My overeating got in the way of me taking care of my family or doing household chores	Once a week	6.0	(4.4–8.2)	14.9	(11.9–18.6)	<0.001
I spent a lot of time feeling sluggish or tired from overeating	2–3 times a week	7.0	(5.1–9.5)	11.3	(8.8–14.5)	0.017
I avoided work, school or social activities because I was afraid I would overeat	Once a month	4.6	(3.2–6.6)	5.2	(3.5–7.6)	0.644
I was so distracted by eating that I could have been hurt (e.g. when driving a car)	Once a month	3.4	(2.2–5.3)	2.4	(1.5–3.9)	0.327
I ate to the point where I felt physically ill	Once a week	2.5	(1.5–4.1)	3.3	(2.0–5.3)	0.410
**Distress and impairment**						
My eating behavior caused me a lot of distress	2–3 times a week	5.8	(4.3–7.9)	17.9	(14.4–21.9)	<0.001
I had significant problems in my life because of food and eating. These may have been problems with my daily routine, work, school, friends, family or health	2–3 times a week	5.6	(3.8–8.0)	12.0	(9.2–15.5)	<0.001
Food addiction measured by mYFAS 2.0		7.5	(5.6–10.0)	16.9	(13.8–20.7)	<0.001

*Note*: The modified Yale Food Addiction Scale 2.0 (mYFAS 2.0) instructs participants to think about common ultra‐processed foods (e.g. sweets, salty snacks, sugary drinks) and other foods that they may have had difficulty with in the past year when considering ‘certain foods’ [37].

### Gender‐stratified prevalence of UPFA by participant demographics and predictor variables

The gender‐stratified associations of participant demographic characteristics and predictor variables with UPFA are listed in Table 3. For both men and women, UPFA was more common among 50–64 year olds than among 65–80 year olds. Household income was significantly associated with UPFA status for women, but not for men. UPFA was higher for women with incomes below $30,000 relative to those with higher incomes. The prevalence of UPFA differed significantly for all predictor variables (e.g. physical health, mental health and social isolation) (Table 3).

**TABLE 3:** Gender‐stratified association of participant demographics and predictor variables with ultra‐processed food addiction (UPFA).

	Prevalence of food addiction			
	Men (*n* = 994)		Women (*n* = 1044)	
	%	95% CI	%	95% CI
Demographics				
Age – 2 categories				
50–64 years (*n* = 1006)	10.3***	(7.4–14.3)	21.0**	(16.0–27.0)
65–80 years (*n* = 1032)	3.7	(2.1–6.3)	12.0	(8.9–16.1)
Race/ethnicity				
White, non‐Hispanic (*n* = 1508)	7.3	(5.3–10.0)	17.3	(13.5–21.9)
Black, non‐Hispanic (*n* = 224)	11.9	(4.2–29.6)	10.5	(5.2–20.0)
Hispanic (*n* = 239)	7.9	(3.4–17.0)	18.9	(11.6–29.3)
Other, non‐Hispanic (*n* = 67)	3.7	(0.7–16.7)	21.3	(7.7–46.7)
Education				
High school or less (*n* = 449)	7.1	(3.7–13.2)	19.4	(13.6–26.9)
Some college (*n* = 895)	7.8	(5.4–11.2)	13.4	(9.5–18.6)
Bachelor’s degree or higher (*n* = 694)	7.8	(5.2–11.7)	16.5	(12.0–22.2)
Four‐level household income				
Less than $30,000 (*n* = 396)	5.4	(2.7–10.3)	24.6*	(17.7–33.2)
$30,000 to <$60,000 (*n* = 570)	7.1	(3.8–12.8)	11.9	(7.5–18.2)
$60,000 to <$100,000 (*n* = 539)	8.2	(4.2–15.3)	18.7	(12.1–27.9)
$100,000 or more (*n* = 533)	8.6	(5.5–13.2)	13.0	(8.5–19.5)
Predictor variables				
How would you describe your weight?*				
Underweight (*n* = 18)	15.9	(2.1–62.2)	53.8***	(23.4–81.5)
Slightly underweight (*n* = 101)	13.8***	(4.3–36.1)	6.1***	(0.9–32.3)
About the right weight (*n* = 530)	0.9	(0.3–2.9)	2.5	(1.0–6.1)
Slightly overweight (*n* = 821)	4.7	(2.5–8.7)	12.9***	(8.8–18.5)
Overweight (*n* = 564)	16.7***	(12.1–22.7)	32.7***	(25.4–41.1)
Physical health				
Excellent/very good/good (*n* = 1589)	5.5	(3.6–8.2)	13.9	(10.6–18.1)
Fair or poor (*n* = 432)	13.6**	(8.9–20.1)	30.1***	(21.9–39.7)
Mental health				
Excellent/very good/good (*n* = 1781)	5.5	(3.8–8.0)	13.7	(10.6–17.6)
Fair or poor (*n* = 219)	22.9***	(14.3–34.6)	44.7***	(31.6–58.7)
In the past year, how often have you felt isolated from others?				
Hardly ever (*n* = 1257)	4.2	(2.5–7.2)	7.5	(5.1–10.9)
Some of the time/often (*n* = 761)	14.1***	(10.0–19.4)	29.2***	(23.1–36.1)

*Note*: For self‐reported weight status, the significant differences across groups are as follows: for women, underweight was significantly different compared with about the right weight; slightly overweight was significantly different compared with about the right weight; overweight was significantly different compared with about the right weight; slightly overweight was significantly different compared with underweight; overweight was significantly different compared with slightly overweight; for men, slightly underweight was significantly different compared with about the right weight; overweight was significantly different compared with about the right weight; overweight was significantly different compared with slightly overweight.

* *P* < 0.05,

** *P* < 0.01,

*** *P* < 0.001.

### Gender‐stratified associations of physical health, mental health and social isolation with UPFA

The PRs of the association between all predictor variables and UPFA are reported in Table 4. All unadjusted and adjusted associations were significant for both older men and women, with medium to large effect sizes, except for the association of self‐reported slightly underweight with UPFA in women. The magnitude of adjusted associations ranged from a PR of 19.86 (95% CI = 7.21–54.71) for the association of underweight with UPFA in women to 1.93 (95% CI = 1.26–2.98) for the association of self‐reported fair/poor physical health with UPFA in women.

**TABLE 4:** Prevalence ratios of the association between all predictor variables and ultra‐processed food addiction (UPFA).

Models^a^	Men						Women					
	Unadjusted			Adjusted^b^			Unadjusted			Adjusted^b^		
	RR	95% CI	*P*	RR	95% CI	*P*	RR	95% CI	*P*	RR	95% CI	*P*
How would you describe your weight?												
Underweight	18.23	(2.03–163.44)	0.010	18.98	(2.55–141.01)	0.004	21.09	(7.11–62.54)	<0.001	19.86	(7.21–54.71)	<0.001
Slightly underweight	15.78	(3.07–81.22)	0.001	13.83	(3.26–58.65)	<0.001	2.39	(0.3–19.04)	0.411	2.39	(0.3–19.26)	0.412
About the right weight												
Slightly overweight	5.40	(1.37–21.37)	0.016	5.32	(1.36–20.8)	0.016	5.06	(1.92–13.33)	0.001	4.90	(1.85–12.95)	0.001
Overweight	19.14	(5.42–67.65)	<0.001	19.14	(5.26–69.66)	<0.001	12.84	(5.08–32.44)	<0.001	11.44	(4.56–28.71)	<0.001
Physical health												
Excellent/very good/good												
Fair or poor	2.48	(1.39–4.41)	0.002	2.99	(1.7–5.26)	<0.001	2.16	(1.45–3.23)	<0.001	1.93	(1.26–2.98)	0.003
Mental health												
Excellent/very good/good												
Fair or poor	4.14	(2.32–7.39)	<0.001	4.02	(2.19–7.38)	<0.001	3.26	(2.18–4.87)	<0.001	2.78	(1.79–4.32)	<0.001
In the past year, how often have you felt isolated from others?												
Hardly ever												
Some of the time/often	3.32	(1.76–6.25)	<0.001	3.35	(1.83–6.14)	<0.001	3.88	(2.5–6.01)	<0.001	3.40	(2.16–5.34)	<0.001

^a^ Each predictor is a separate model.

^b^ Adjusted for age, race and ethnicity, education, and income.

## DISCUSSION

In a nationally representative sample of adults aged 50–80 years old, the overall prevalence rate for UPFA was found to be 12.4%. This is similar to the prevalence of UPFA of 14% for non‐clinical samples of adults found in a recent meta‐analysis of over 200 studies [12]. This estimate is higher than the other study that investigated UPFA specifically among older women (prevalence of 5.8%) [29]. However, the prior study utilized an older measure of UPFA that may underestimate its prevalence [12] and was not based on a nationally representative sample [29]. Thus, the current study likely provides a more accurate estimate of the level of UPFA in older adults in the US. The estimated prevalence of UPFA in the current sample is much higher when compared with the prevalence rates of AUD (1.5%) and TUD (4%) among older adults [34].

In the current study, there also appear to be cohort effects in the prevalence of UPFA. Adults aged 50–64 years (15.7%) had almost double the prevalence of UPFA than adults aged 65–80 years (8.2%). Similarly, AUD and TUD rates have been found to be higher in those aged 50–64 years relative to those aged 65–80 years [34]. The changing nutrient quality and food environment in the 1970s and 1980s may have played a contributing role in the heightened level of UPFA in the younger cohort (15.7%; 50–64 years old) relative to the older cohort (8.2%; 65–80 years old). The younger cohort were children and teens in the decades when exposure to UPF was increasing, whereas the older cohort was in their 20s and 30s. Exposure to addictive substances earlier in development has been associated with an increased risk of developing a future SUD [52, 53]. Delaying regular exposure to addictive substances until the age of 25 years or older is associated with a substantially reduced likelihood of developing an addiction to that substance [54]. Thus, the younger cohort of older adults in this sample may have been in a higher‐risk developmental period when environmental exposure to UPF increased on a population level [4], potentially contributing to a higher likelihood of UPFA later in life. In future research, it will be important to evaluate whether there are critical developmental risk periods where UPF exposure may be more likely to increase risk for future addictive patterns of intake [55]. Further, this cohort of older adults is the first to spend the majority of their lifespan in a food environment dominated by UPFs. Currently, UPFs comprise the majority of calories for children and adolescents in the USA [56]. It is plausible that the rates of UPFA in older adulthood will be even higher for future generations who have spent the entirety of their life in a food environment dominated by UPFs.

Older adult women (16.9%) had a higher rate of UPFA relative to older adult men (7.6%). Women aged 50–64 years had the highest endorsement of UPFA, with 21% meeting this criterion. While UPFA was more prevalent among older women, traditional SUDs are typically more common in older men. For example, women aged 65 years and older display lower 12‐month prevalence rates of AUD (0.5%) and TUD (3.7%) compared with men in the same age group (2.8% AUD and 4.5% TUD) [34]. Lower SUD rates in women have been linked, in part, to historically higher levels of social disapproval of substance use in women [57, 58]. However, as the social acceptability for alcohol and tobacco use has increased for women, the rates of these SUDs in women are becoming more similar to those seen in men [58, 59]. In contrast, the access to and marketing of UPFs is highly prevalent for both women and men [3, 60, 61]. In fact, older women may have had greater exposure to UPFs than older men during critical developmental periods. In the 1980s, public health efforts to reduce fat intake led to a surge in high‐carbohydrate, low‐fat UPFs marketed as diet options, such as low‐fat cookies and microwavable meals [62]. These foods, with marketing often aimed at women, were believed to aid in weight management, but current research indicates that these UPFs may be addictive in part because of their high levels of refined carbohydrates [10, 62, 63]. Women aged 50–64 years today were in a potentially vulnerable developmental period during the rise of these diet UPFs, perhaps consuming them under the misconception that they would aid in weight loss while actually reinforcing addictive eating patterns. These factors may have converged to contribute to the particularly high level of UPFA (21%) for women aged 50–64 years in the current study. Adolescent girls and young women are still major targets for diet UPFs today (particularly through social media) [64], which warrants further exploration in relation to the development of UPFA.

In this sample of older adults, UPFA was associated with lower perceived physical, mental and social well‐being across both genders. Weight status is a potential marker of physical health. Older adult men who reported being overweight were 19.14 times more likely to meet the criteria for UFPA relative to those who reported being at about the right weight, while older adult women who reported being overweight were 11.44 times more likely to meet the criteria for UPFA. There is a consistent association with overweight and obesity with UPFA in younger samples [12, 17, 18]. The magnitude of this association is also similar to that found between class‐2 obesity (BMI ≥ 35.0 kg/m^2^) and UPFA for women nurses in the only other study of UPFA in older adults (PR = 15.83 for women aged 45–64 years; PR = 18.41 for women aged 62–88 years) [29]. The current study findings suggest that overweight and UPFA are more strongly associated in older men than in older women. There was also an unexpected association between perceptions of being underweight and higher UPFA in both genders. These findings should be interpreted with caution as those who reported being underweight were a small subset of the sample (*n* = 18) and the confidence intervals are large, which suggests great variability. Future research is needed to examine the association of UPFA with objective measures of weight, BMI and body composition.

Alongside weight status, perceptions of poorer physical health were also associated with UPFA in both genders. Older men in this study who reported fair or poor physical health were 2.99 times more likely to meet the criteria for UPFA than those who did not. Older adult women who reported fair or poor physical health were 1.93 times more likely to meet the criteria for UPFA. These findings are similar to the magnitude of the co‐occurrence between SUDs and physical health conditions in older adults [65]. Further research is needed to understand what specific medical conditions may be driving endorsements of poor physical health in older adults. Prior research has linked UPFA with hypercholesterolemia and type‐2 diabetes [20, 29]. Research investigating the association of UPFA and specific medical conditions using medical records will be an important next step.

In this older adult sample, men who reported fair or poor mental health were 4.02 times more likely to meet the criteria for UPFA, while women with fair or poor mental health were 2.78 times more likely. This finding is consistent with other studies in non‐older adult populations, finding an association between UPFA and a range of mental health concerns, such as anxiety, depression, post‐traumatic stress disorder and severe mental illness [21, 66, 67]. Individuals with mental health concerns may be more likely to consume UPFs as a strategy to cope with emotional distress, which may lead to increased vulnerability for the development of UPFA [23, 68, 69, 70]. There is also growing evidence that greater levels of UPF intake are predictive of worsening mental health [71, 72, 73], potentially through pathways including inflammation, gut‐microbiome dysfunction, and negative feelings of shame and guilt about overeating [74, 75, 76, 77]. Future research is needed to understand the complex, bi‐directional associations between UPF intake, UPFA and mental health functioning in older adults.

Older men who reported feeling socially isolated in the past year were 3.35 times more likely to meet the criteria for UPFA. Women in this study who reported feeling socially isolated in the past year were 3.40 times more likely to meet the criteria for UPFA. Social isolation is becoming an increasingly prevalent public health concern [78]. Social isolation has been associated with worse physical health (e.g. coronary heart disease) and a variety of mental health concerns (e.g. cognitive decline, substance use, depression, increased psychological distress) [79, 80, 81]. There may be multiple pathways linking UPFA with social isolation. Older adults may consume UPFs to cope with negative effects associated with social isolation, which could then increase the risk of developing UPFA. Further, UPFA could also plausibly increase the likelihood of social isolation. Individuals with UPFA report socially isolating themselves to avoid others from seeing how much they eat [76], which may weaken social networks over time. Future research, particularly longitudinal studies, is needed to understand the mechanisms underlying the association between social isolation and UPFA.

The current study has several strengths. First, the study used a nationally representative sample of older adults. Second, the preliminary associations of UPFA were investigated across physical, mental and social well‐being domains. Third, this study was sufficiently powered to examine gender differences, which is important given the lack of prior research on UPFA in older men. However, there are also some limitations to the current study. Mainly, this study was a cross‐sectional observational study, which limits the ability to make causal inferences. Additionally, data for the current study were collected during the COVID‐19 pandemic. By July of 2022, vaccines against COVID‐19 had been developed and distributed to the public. Individuals, especially older adults, may have been practicing social distancing to remain healthy and avoid serious health complications related to COVID‐19. This could have led to an increase in self‐reported feelings of loneliness in the past year in the current study. Additionally, research has shown that during the COVID‐19 pandemic individuals reported snacking more often and consumed more UPFs, such as sweets [82], which may have impacted their eating patterns and risk for UPFA. Future longitudinal research is needed to further understand the temporal associations of UPFA with deleterious physical, mental and social well‐being outcomes. Additionally, one‐item measures of health and functioning were used to reduce participant survey burden. Future research should implement more robust measures of physical health, mental health and social well‐being to more closely examine the association of these factors with UPFA. Future research should be conducted using robust dietary quality measures, such as 24‐hour dietary recalls, to assess aspects of diet quality and UPFA in older adults. Further research should collect objective measures of height, weight and BMI in addition to objective measures of body composition, such as dual energy X‐ray absorptiometry (DEXA), to assess changes in muscle mass, body fat and frailty, which may be of particular importance to older adults.

## CONCLUSION

The findings presented are the first to our knowledge that examine UPFA prevalence rates in a nationally representative sample of older adults. There is evidence that UPFA is an overlooked condition impacting older adults, with an overall prevalence rate of 12.4% and marked gender and age cohort differences. Of note, one in five women aged 50–64 years met the criteria for UPFA. The current study provides preliminary support that UPFA is associated with poorer physical health, mental health and social well‐being. Further research should be conducted to investigate the developmental course and lifespan impacts of UPFA given that the current food environment is saturated with UPFs.

## AUTHOR CONTRIBUTIONS

**Lucy K. Loch:** Writing—original draft (lead); writing—review and editing (lead). **Matthias Kirch:** Formal analysis (lead); writing—review and editing (supporting). **Dianne C. Singer:** Data curation (lead); project administration (lead); writing—review and editing (supporting). **Erica Solway:** Conceptualization (supporting); data curation (supporting); project administration (supporting); writing—review and editing (supporting). **J. Scott Roberts:** Conceptualization (supporting); data curation (supporting); project administration (supporting); writing—review and editing (supporting). **Jeffrey T. Kullgren:** Conceptualization (supporting); data curation (supporting); project administration (supporting); writing—review and editing (supporting). **Ashley N. Gearhardt:** Conceptualization (supporting); supervision (supporting); writing—original draft (supporting); writing—review and editing (supporting).

## DECLARATION OF INTERESTS

None to declare.

## Supporting information


**Table S1.** Sample demographics, non‐gender stratified.
**Table S2.** Response frequencies meeting ultra‐processed food addiction (UPFA) diagnostic criteria measured by mYFAS 2.0, non‐gender stratified.
**Table S3.** Association of participant demographics and predictor variables with ultra‐processed food addiction (UPFA), non‐gender stratified.
**Table S4.** Prevalence ratios of the association between all predictor variables and ultra‐processed food addiction (UPFA), non‐gender stratified.

## Data Availability

The data for the current project is not publicly available. The National Poll on Health Aging releases waves of data approximately 18‐24 months after fielding.
