# Supplementary material for: Ultra‐processed food addiction in a nationally representative sample of older adults in the USA
Source: Addiction. 2025 Sep 29;121(3):510–21. doi: 10.1111/add.70186 (PMC12887916; doi:10.1111/add.70186)
Supplement: Supplementary file 1 — Table S1. Sample demographics, non‐gender stratified. Table S2. Response frequencies meeting ultra‐processed food addiction (UPFA) diagnostic criteria measured by mYFAS 2.0, non‐gender stratified. Table S3. Association of participant demographics and predictor variables with ultra‐processed food addiction (UPFA), non‐gender stratified. Table S4. Prevalence ratios of the association between all predictor variables and ultra‐processed food addiction (UPFA), non‐gender stratified. [file ADD-121-510-s001.docx]

Supplementary Material

**Table 1**

*Sample demographics, non-gender stratified*

|  | **US Adults age 50-80 (N=2,038)** | |
| --- | --- | --- |
|  | **Weighted %** | **95% CI** |
| **Demographic Variables** |  |  |
| Age - 2 Categories |  |  |
| 50-64 | 56.4 | [53.5, 59.2] |
| 65-80 | 43.6 | [40.8, 46.5] |
| Respondent gender |  |  |
| Male | 48.4 | [45.5, 51.4] |
| Female | 51.6 | [48.6, 54.5] |
| Race/Ethnicity |  |  |
| White, Non-Hispanic | 69.7 | [66.7, 72.6] |
| Black, Non-Hispanic | 10.9 | [9.1, 13.0] |
| Hispanic | 12.5 | [10.8, 14.6] |
| Other, Non-Hispanic | 6.9 | [5.0, 9.3] |
| Education |  |  |
| High school or less | 39.5 | [36.4, 42.7] |
| Some college | 25.3 | [23.3, 27.4] |
| Bachelor's degree or higher | 35.2 | [32.5, 38.0] |
| 4-level Household Income |  |  |
| Less than $30,000 | 20.5 | [18.1, 23.1] |
| $30,000 to under $60,000 | 25.2 | [22.8, 27.7] |
| $60,000 to under $100,000 | 26.9 | [24.3, 29.6] |
| $100,000 or more | 27.5 | [24.9, 30.2] |
| **Predictor Variables** |  |  |
| How would you describe your weight? |  |  |
| About the right weight | 25.5 | [23.0, 28.1] |
| Underweight | 1.0 | [0.6, 1.8] |
| Slightly underweight | 5.3 | [4.1, 6.8] |
| Slightly overweight | 39.5 | [36.6, 42.4] |
| Overweight | 28.7 | [26.1, 31.5] |
| Physical health |  |  |
| Excellent/V good/Good | 78.1 | [75.5, 80.4] |
| Fair or poor | 21.9 | [19.6, 24.5] |
| Mental health |  |  |
| Excellent/V good/Good | 88.9 | [86.8, 90.7] |
| Fair or poor | 11.1 | [9.3, 13.2] |
| In the past year, how often have you felt isolated from others? |  |  |
| Hardly ever | 61.5 | [58.5, 64.4] |
| Some of the time/often | 38.5 | [35.6, 41.5] |

**Table 2**

*Response frequencies meeting UPFA diagnostic criteria measured by the mYFAS 2.0, non-gender stratified*

| **Symptom** | **Threshold** | **Adults age 50-80 meeting threshold** | |
| --- | --- | --- | --- |
|  |  | **Proportion** | **95% CI** |
| I had such strong urges to eat certain foods that I couldn't think of anything else. | Once a week | 23.8 | [21.2, 26.6] |
| I tried and failed to cut down on or stop eating certain foods. | 2-3 times a week | 19.6 | [17.2, 22.3] |
| If I had emotional problems because I hadn't eaten certain foods, I would eat them. | Once a week | 17.1 | [14.9, 19.5] |
| Eating the same amount of food did not give me as much enjoyment as it used to. | 2-3 times a week | 13.0 | [11.0, 15.3] |
| I kept eating in the same way even though my eating caused emotional problems. | Once a month | 11.4 | [9.5, 13.6] |
| My friends or family were worried about how much I overate. | Once a month | 10.1 | [8.4, 12.2] |
| My overeating got in the way of me taking care of my family or doing household chores. | Once a week | 10.6 | [8.8, 12.8] |
| I spent a lot of time feeling sluggish or tired from overeating. | 2-3 times a week | 9.3 | [7.6, 11.2] |
| I avoided work, school or social activities because I was afraid I would overeat. | Once a month | 4.9 | [3.7, 6.4] |
| I was so distracted by eating that I could have been hurt (e.g., when driving a car). | Once a month | 2.9 | [2.1, 4.0] |
| I ate to the point where I felt physically ill. | Once a week | 2.9 | [2.0, 4.1] |
| **Distress and impairment** |  |  |  |
| My eating behavior caused me a lot of distress. | 2-3 times a week | 12.0 | [10.0, 14.4] |
| I had significant problems in my life because of food and eating. These may have been problems with my daily routine, work, school, friends, family, or health. | 2-3 times a week | 8.9 | [7.1, 11.0] |
| Food addiction by using the mYFAS 2.0 |  | 12.4 | [10.4, 14.7] |

*Note: The mYFAS 2.0 instructs participants to think about common UPFs (e.g., sweets, salty snacks, sugary drinks) and other foods they may have had difficulty with in the past year when considering “certain foods.”^37^*

**Table 3**

*Association of participant demographics and predictor variables with UPFA, non-gender stratified*

|  | **Prevalence of food addiction** | |
| --- | --- | --- |
|  | **%** | **95% CI** |
| Demographics |  |  |
| Age - 2 Categories |  |  |
| 50-64 (n=1006) | 15.7*** | [12.6, 19.3] |
| 65-80 (n=1032) | 8.2 | [6.2, 10.6] |
| Race/Ethnicity |  |  |
| White, Non-Hispanic (n=1508) | 12.4 | [10.1, 15.1] |
| Black, Non-Hispanic (n=224) | 11.1 | [6.1, 19.3] |
| Hispanic (n=239) | 13.6 | [8.9, 20.2] |
| Other, Non-Hispanic (n=67) | 12.2 | [5.0, 26.9] |
| Education |  |  |
| High school or less (n=449) | 13.8 | [10.1, 18.7] |
| Some college (n=895) | 10.7 | [8.2, 13.7] |
| Bachelor's degree or higher (n=694) | 12.0 | [9.3, 15.4] |
| 4-level Household Income |  |  |
| Less than $30,000 (n=396) | 16.1 | [11.8, 21.5] |
| $30,000 to under $60,000 (n=570) | 9.7 | [6.7, 13.9] |
| $60,000 to under $100,000 (n=539) | 14.1 | [9.7, 20.0] |
| $100,000 or more (n=533) | 10.4 | [7.7, 14.0] |
| Predictor Variables |  |  |
| How would you describe your weight? |  |  |
| About the right weight (n=530) | 1.7 | [0.8, 3.5] |
| Underweight (n=18) | 32.0*** | [13.0, 59.7] |
| Slightly underweight (n=101) | 11.1*** | [4.0, 26.9] |
| Slightly overweight (n=821) | 9.0*** | [6.5, 12.4] |
| Overweight (n=564) | 25.7*** | [20.9, 31.3] |
| Physical health |  |  |
| Excellent/V good/Good (n=1589) | 10.0 | [7.9, 12.5] |
| Fair or poor (n=432) | 21.0*** | [16.3, 26.7] |
| Mental health |  |  |
| Excellent/V good/Good (n=1781) | 9.8 | [7.9, 12.1] |
| Fair or poor (n=219) | 33.0*** | [24.8, 42.3] |
| In the past year, how often have you felt isolated from others? |  |  |
| Hardly ever (n=1257) | 5.8 | [4.2, 7.9] |
| Some of the time/often (n=761) | 22.8*** | [18.7, 27.6] |
| *** p-value from Pearson's chi-2 <0.001 |  |  |

**Note: For self-reported weight status significant differences across groups are as follows:*

*Underweight significantly different from about the right weight, slightly underweight significantly different from about the right weight, slightly overweight significantly different from about the right weight, overweight significantly different from about the right weight*

**Table 4**

*Prevalence ratios of the association between all predictor variables and UPFA, non-gender stratified*

|  | **US adults age 50-80** | | | | | |
| --- | --- | --- | --- | --- | --- | --- |
|  | **Unadjusted** | | | **Adjusted**** | | |
| **Models*** | **Risk Ratio** | **95% CI** | **p-value** | **Risk Ratio** | **95% CI** | **p-value** |
| How would you describe your weight? |  |  |  |  |  |  |
| Underweight | 19.00 | [6.51, 55.42] | <0.001 | 17.29 | [5.86, 50.98] | <0.001 |
| Slightly underweight | 6.57 | [1.96, 22.08] | 0.002 | 6.47 | [2.01, 20.8] | 0.002 |
| About the right weight |  |  |  |  |  |  |
| Slightly overweight | 5.33 | [2.39, 11.9] | <0.001 | 5.26 | [2.36, 11.7] | <0.001 |
| Overweight | 15.26 | [7.13, 32.68] | <0.001 | 14.22 | [6.64, 30.45] | <0.001 |
| Physical health |  |  |  |  |  |  |
| Excellent/V good/Good |  |  |  |  |  |  |
| Fair or poor | 2.11 | [1.5, 2.95] | <0.001 | 2.11 | [1.49, 2.99] | <0.001 |
| Mental health |  |  |  |  |  |  |
| Excellent/V good/Good |  |  |  |  |  |  |
| Fair or poor | 3.37 | [2.4, 4.74] | <0.001 | 3.25 | [2.31, 4.56] | <0.001 |
| In the past year, how often have you felt isolated from others? |  |  |  |  |  |  |
| Hardly ever |  |  |  |  |  |  |
| Some of the time/often | 3.94 | [2.73, 5.69] | <0.001 | 3.68 | [2.56, 5.29] | <0.001 |

| * Each predictor is a separate model. |
| --- |
| ** Adjusted for age, race and ethnicity, education, and income |
